# Mesenchymal Stem Cell Secretome: Toward Cell-Free Therapeutic Strategies in Regenerative Medicine

**DOI:** 10.3390/ijms18091852

**Published:** 2017-08-25

**Authors:** Francisco J. Vizoso, Noemi Eiro, Sandra Cid, Jose Schneider, Roman Perez-Fernandez

**Affiliations:** 1 Research Unit, Fundación Hospital de Jove, Avda. Eduardo Castro, 161, 33290 Gijón, Spain; noemi.eiro@gmail.com (N.E.); investigacion@hospitaldejove.com (S.C.); 2 Department of Obstetrics & Gynecology, C/Ramon y Cajal 7, University of Valladolid, 47005 Valladolid, Spain; jose.schneider@urjc.es; 3 Department of Physiology-Center for Research in Molecular Medicine and Chronic Diseases (CIMUS), University of Santiago de Compostela, 15706 Santiago de Compostela, Spain

**Keywords:** conditioned media, exosomes, mesenchymal stem cells, adipose-derived stem cells, bone marrow mesenchymal stem cells, uterine cervical stem cells, hUCESCs

## Abstract

Earlier research primarily attributed the effects of mesenchymal stem cell (MSC) therapies to their capacity for local engrafting and differentiating into multiple tissue types. However, recent studies have revealed that implanted cells do not survive for long, and that the benefits of MSC therapy could be due to the vast array of bioactive factors they produce, which play an important role in the regulation of key biologic processes. Secretome derivatives, such as conditioned media or exosomes, may present considerable advantages over cells for manufacturing, storage, handling, product shelf life and their potential as a ready-to-go biologic product. Nevertheless, regulatory requirements for manufacturing and quality control will be necessary to establish the safety and efficacy profile of these products. Among MSCs, human uterine cervical stem cells (hUCESCs) may be a good candidate for obtaining secretome-derived products. hUCESCs are obtained by Pap cervical smear, which is a less invasive and painful method than those used for obtaining other MSCs (for example, from bone marrow or adipose tissue). Moreover, due to easy isolation and a high proliferative rate, it is possible to obtain large amounts of hUCESCs or secretome-derived products for research and clinical use.

## 1. Stem Cells and Regenerative Medicine

Stem cells are immature tissue precursor cells that are able to self-renew, have the ability to form clonal cell populations, and differentiate into multiple cell lineages [1,2]. These special properties are particularly attractive for restoring function in multiple tissues. Currently, the broad diversity of stem cells can be divided into three categories: (i) embryonic stem cells derived from early stage embryos; (ii) induced pluripotent stem cells and (iii) adult stem cells including hematopoietic stem cells, neural stem cells and mesenchymal stem cells.

The therapeutic potential of stem cells can be attributed to three key mechanisms of action. The first is “homing”, for which systemically administered stem cells migrate to the focus of acute injury due to chemical gradients. It is hypothesized that migration to target tissues occurs through a process similar to that of leukocyte migration. Chemoattraction is mediated by cell surface receptors such as the chemokine receptors. Although the exact mechanism of stem cell-endothelial interaction at the target site is not well established, integrins and selectins mediate these interactions [3,4]. Transmigration of stem cells to the focus of injury occurs across the endothelium via leukocyte-like pathways involving vascular cell adhesion molecule 1 (VCAM-1) and G-protein-coupled receptor signaling [5]. The second mechanism is differentiation into multiple cell types, which locally engraft and induce restoration of function by augmenting or replacing damaged tissues [6,7]. The third major mechanism is secretion of bioactive factors, which may potentially affect both local and systemic physiological processes [8].

Adult stem cells guarantee the maintenance and repair of adult tissues and organs. Mesenchymal stem cells (MSCs) are especially emerging as cell-based therapy of numerous diseases.

## 2. Mesenchymal Stem Cells (MSCs)

Adult bone marrow MSCs (BMMSCs) were discovered in 1968 by Friedenstein et al. [9], who described a fibroblast-like population able to secrete growth factors and cytokines relevant for hematopoiesis and others processes. MSCs have an apparently ubiquitous localization. They have also been isolated from other adult tissues such as adipose tissue [10], skin [11], lung [12], synovial membrane [13], dental pulp [14], nasal olfactory mucosa [15], breast milk [16], scalp tissue [17], muscle [18], periosteum [19], corneal limbus [20], peripheral blood [21], endometrial and menstrual blood [22], and uterine cervix [23], as well as from fetal/neonatal tissues [24,25,26,27]. All of these MCSs can be isolated and expanded from stroma of many tissues, the bone marrow and subcutaneous fat being the preferred sources [28]. The Mesenchymal and Tissue Stem Cell Committee of the International Society for Cellular Therapy established in 2006 the minimal identifying characteristics for human MSCs [29]: (a) plastic-adherent cells when maintained in standard culture conditions; (b) expression of CD105, CD73 and CD90, and lack expression of CD45, CD34, CD14 or CD11b, CD79a or CD19 and HLA-DR surface molecules and (c) capacity to differentiate into osteoblasts, adipocytes and chondroblasts in vitro [29].

Although MSCs are present in multiple tissues, their overall quantity in the body is scarce. Cell therapy protocols generally require hundreds of millions of MSCs per treatment; therefore, cell expansion in vitro is needed for about 10 weeks before implantation. The patient’s age and clinical characteristics influence the optimal culture conditions for clinical scale production of human MSCs [30,31]. The cellular senescence of MSCs in vitro contributes to aging and age-related diseases [32]. 

Earlier research attributed the therapeutic effects of MSCs to their engrafting and differentiation capacity. However, several studies have revealed that the implantation time of MSCs is usually too short to have an effective impact [33,34,35,36,37]. Indeed, it has been reported that <1% MSCs survive for more than one week after systemic administration [38,39,40,41,42], suggesting that the main effects of MSCs are probably mediated by paracrine mechanisms [43]. Recent studies have also brought attention to the wide array of bioactive factors produced by MSCs, which may play an important role in the regulation of numerous physiological processes [44]. Therefore, the secretome from MSCs has attracted much attention for its potential use in tissue repair and regeneration [43,45,46,47].

## 3. Secretome and Conditioned Media from MSCs as New Therapeutic Strategy

We are presently witnessing the emergence of a novel type of biological regulation involving the communication between cells via their secreted substances, the secretome. The secretome is defined as the set of factors/molecules secreted to the extracellular space. These factors include, among others, soluble proteins, free nucleic acids, lipids and extracellular vesicles. The latter can be subdivided into apoptotic bodies, microparticles and exosomes [48]. The secretome of individual cells and tissues is specific, and changes in response to fluctuations in physiological states or pathological conditions.

The use of cell-free therapies such as MSC-sourced secretome in regenerative medicine provides key advantages over stem-cell based applications: (a) application of the secretome resolves several safety considerations potentially associated with the transplantation of living and proliferative cell populations including immune compatibility, tumorigenicity, emboli formation and the transmission of infections; (b) MSC-sourced secretome may be evaluated for safety, dosage and potency in a manner analogous to conventional pharmaceutical agents; (c) storage can be done without application of potentially toxic cryopreservative agents for a long period without loss of product potency [23,49,50]; (d) using MSC-sourced secretome, such as conditioned medium (CM), is more economical and more practical for clinical application since it avoids invasive cell collection procedures [51]; (e) mass-production is possible through tailor-made cell lines under controlled laboratory conditions, providing a convenient source of bioactive factors; (f) the time and cost of expansion and maintenance of cultured stem cells could be greatly reduced and off-the-shelf secretome therapies could be immediately available for treatment of acute conditions such as cerebral ischemia, myocardial infarction, or military trauma; and (g) finally, the biological product obtained for therapeutic applications could be modified to desired cell-specific effects.

CM represents the complete regenerative milieu of cell-sourced secretome and vesicular elements. The soluble components of the secretome may be separated from the microvesicle fraction by centrifugation, filtration, polymer precipitation-based methodologies, ion exchange chromatography and size-exclusion chromatography [52,53]. Both of these components may be capable of independently triggering regeneration and repair as well as of mediating the de novo organogenesis of tissue-engineered organs ex vivo [43,54].

It has been demonstrated that MSC-derived CM is sufficient to significantly improve multiple biomarkers of pathophysiology, and, in general, to be as effective as transplantation of the corresponding MSCs in a long list of animal models (Table 1).

## 4. MSC-Secretome Mechanisms and Applications

### 4.1. Immunomodulation and Antiinflammatory Activity

MSCs affect the proliferation, activation and function of immune cells. Pre-clinical studies on animal models have shown a suppressive effect on both innate and adaptive immunity [72,73]. In addition, there is extensive clinical experience on MSCs based on interventional clinical studies (http://clinicaltrials.gov). The most promising potential applications involve the treatment of graft versus host disease [74,75], as well as of autoimmune and inflammatory diseases, such as insulin systemic lupus erythematosus (SLE), diabetes mellitus type I, (SLE), multiple sclerosis or Crohn’s disease [73]. Interestingly, MSCs typically express major histocompatibility complex (MHC) -I but lack expression of MHC-II, CD40, CD80, and CD86 on the cell surface, and thus they escape T cell recognition and often fail to induce an immune response by the transplant host [76]. It has been widely shown that MSCs are capable of affecting activation and proliferation of all immune cell types [77]. Di Nicola et al. found that MSCs inhibit proliferation of CD4^+^ and CD8^+^ T cells [78]. In addition, MSCs act in three major stages of immune response: antigen recognition and presentation; T cell activation, proliferation, and differentiation; and the T-cell effector stage [79]. Our studies have also demonstrated an inhibitory effect of the CM from human uterine cervical stem cells (hUCESC-CM) on monocyte–macrophage differentiation as well as on macrophage–monocyte de-differentiation [25]. 

It is well established that the antiinflammatory effect of MSC-CM is at least in part mediated by soluble immunoregulatory molecules. Among the antiinflammatory cytokines present in MSC-CM are tumor necrosis factor β1 (TGFβ1) [63], interleukin (IL) 13 [50,63], IL18 binding protein (IL18BP), ciliary neurotrophic factor (CNTF), neurotrophin 3 (NT-3) factor [50], IL10, IL12p70, IL17E, IL27 or IL1 receptor antagonist (IL1RA) [63]. MSC-CM has also been found to contain pro-inflammatory cytokines, such as IL1b [63], IL6 [80,81], IL8 [82,83] and IL9 [83]. The balance between these anti-inflammatory and pro-inflammatory cytokines may determine the final effect. Nevertheless, it is also remarkable that MSCs inhibit proinflammatory cytokines, such as interferon (IFN)-γ and tumor necrosis factor α (TNFα), while increasing antiinflammatory IL10 release [84,85]. Our recent findings derived from an experimental model of uveitis in rats [50] corroborate this. We found that hUCESC-CM treatment significantly reduced mRNA expression of IL6, IL8, TNFα and MIP-1α pro-inflammatory cytokines, but increased mRNA expression of the IL10 anti-inflammatory cytokine, which is similar to results using MSC-CM from amniotic fluid in a mice colitis model [62]. We also found that hUCESC-CM reduces the infiltration of leucocytes in ocular tissues [52].

### 4.2. Anti-Apoptotic Activity

MSCs prevent cell death via restoration of local microenvironment by producing inhibitor proteins of apoptosis and by decreasing expression of anti-apoptotic proteins [86]. Thus, it was reported that MSCs decrease the pro-apoptotic factors Bax and cleaved caspase-3 expression but increase the anti-apoptotic Bcl-2 levels, while expression of pro-angiogenic factors, such as basic fibroblastic growth factor (bFGF), vascular endothelial growth factor (VEGF), and CXCL12 were increased in MSC-treated hearts compared to medium-treated hearts [87]. It is worth noting that our team has found that hUCESC-CM has a different effect on normal cells than on cancer cells. While an antiapoptotic effect was observed in normal cells [50], we found that hUCESC-CM induced apoptosis in cancer cells both in vitro and in vivo [23].

### 4.3. Wound Healing and Tissue Repair

A beneficial role on wound healing and tissue repair was observed by MSCs at the site of injury [41]. Data from animal models seem to indicate that autocrine or paracrine effects of MSCs rather than their direct engraftment and tissue differentiation may play the key role in wound healing [88,89,90,91]. Recently, we reported the efficacy of hUCESC-CM on epithelial healing in a rat model of dry eyes after alkaline corneal epithelial ulcer induction. After injury, dry eyes treated with hUCESC-CM improved epithelial regeneration and induced reduced corneal MIP-1α and TNF-α mRNA expression [49]. Corneal avascularity is required for optical transparency, so it is relevant that hUCESC-CM treatment did not produce vascularization in this model. Inflammation, chemical burns and corneal infections induce leukocytes to infiltrate the cornea, which induce corneal neovascularization by releasing inflammatory cytokines [92,93].

Several studies have reported the presence of growth factors in MSC-CM that contribute to the regeneration of damaged organ tissues, with special emphasis on proliferation [49,60,63,67,82,83,94,95,96,97,98]. It is also worth mentioning that secretome from MSCs has anti-fibrotic and angiogenic effects that can reduce scar formation [99,100] and improve the long-term ejection fraction when administered early or prior to adverse remodeling in experimental models of myocardial infarction [100,101,102]. 

### 4.4. Neuroprotective and Neurotrophic Effects

Over the past decade, numerous studies have appeared supporting the neuroprotective and neurotrophic effects of MSC-secretome [103,104,105]. In fact, it is known that MSC-CM contains a number of neurothophic factors [80,103,104,105,106,107]. Several studies have reported beneficial effects of MSC-based approaches on nerve injury models. These effects include modulation of the inflammatory environment on the site, enhanced vascularization of the regenerating site, increased thickness of the myelin sheaths, modulation of the Wallerian degeneration stage, accelerated fibre regeneration and increase in the number thereof, reduction of fibrotic scaring, and improved fibre organization [104].

A key role of MSC secretome as a modulator of the neurogenic niche has been recently reported. Both neural stem cells (NSCs) and MSCs secrete a panel of growth factors. Likewise, mutually beneficially effects have been shown when these cell types are co-cultured in vitro [108]. 

Beneficial effects of CM derived from stem cells of human exfoliated deciduous teeth (SHEDs) in an animal model of Alzheimer’s disease have recently been reported. Intranasal administration of SHEDs improved cognitive function and induced neuroregenerative effects such as an attenuated pro-inflammatory response induced by amyloid plaques, and anti-inflammatory M2-like microglia [64]. 

### 4.5. Angiogenesis Regulation

Angiogenesis is defined as the process by which new vasculature sprouts from pre-existing blood vessels. Normal angiogenesis is important during wound healing process. Various studies have demonstrated the effect of MSC secretome on key steps in angiogenesis. For example, different MSC populations (e.g., adipose, amniotic, bone marrow (BM) and Wharton jelly umbilical vein) induce proliferation and migration of endothelial cells promoting tube formation, as well as prevent endothelial cell apoptosis in vitro [109].

The role of MSCs in angiogenesis is of great interest given the large spectrum of clinical diseases related to insufficient or abnormal vessel growth, including atherosclerotic diseases and wound healing disorders. Successful application of MSCs to promote angiogenesis in different animal models of cerebral ischemia/stroke, myocardial infarction, neurogenic bladder, peripheral artery disease, and stress urinary incontinence has been demonstrated [110,111,112].

A number of angiogenic stimulators and inhibitors have been identified in MSC secretome [63,82,113,114,115,116,117,118,119,120,121]. Recently, an extensive proteomic analysis of the MSC-CM stimulated with inflammatory cytokines led to the identification of tissue inhibitor of metalloproteinase-1 (TIMP-1) as the molecule responsible for the antiangiogenic effects of MSCs [122]. All of these data indicate that various factors present in MSC-CM may represent a balanced cocktail that acts in concert to promote angiogenesis. Moreover, several studies demonstrate that the secretion of these pro- and anti-angiogenic factors can be modified depending on chemokines and hypoxic conditions. Thus, TGFα had the ability to increase the level of several growth factors (i.e., VEGF, hepatocyte growth factor (HGF), platelet-derived growth factor (PDGF), IL6 and IL8). Likewise, CM from MSCs treated with TFG-α induces blood vessel growth in an in vivo assay [123].

### 4.6. Antitumor Effect

Various studies have paid attention to the relationship between MSCs and the oncogenic process. Basically, the role of MSCs in cancer could be carried out as: (a) an indirect effect, via modulatory effect of MSCs on tumors; (b) as a direct effect, via malignant transformation of the MSCs themselves [124]. Contradictory results have been described regarding pro- or anti-tumor effect induced by MSCs, both in vitro and in vivo [124,125]. These discrepancies about MSC functionality might be due to the absence of specific markers to isolate more homogeneous population. Contradictory results have been also reported with respect to MSC-CM. For example, BMMSC-CM has either anti-tumor effects on non-small cell lung cancer cells [126] or stimulatory effects on myeloma cells [127], whereas adipose stem cell conditioned medium (ADSC-CM) has no effect on human glioblastoma cancer stem cell subpopulations [128]. Specific anti-tumor effects of hUCESC-CM on proliferation, apoptosis, and tumor-cell invasiveness have been demonstrated. hUCESC-CM inhibits the aggressive behavior of breast cancer cells in vitro (cell lines and primary tumors) and reduces tumor growth in vivo in a mouse xenograft tumor model. These findings differ from those obtained using other types of MSC-CM [129,130,131]. Compared to adipose stem cell conditioned medium (ADSC-CM), hUCESC-CM contains low levels of factors involved in cancer progression, e.g., EGFR, FGF-4 and -9, intracellular adhesion molecule-3, IL6, IL6R, CCL7, macrophage migration inhibitory factor (MIF), soluble gp130 and VEGF-D [132,133,134,135,136,137,138,139]. On the other hand, hUCESC-CM contains high levels of factors involved in inhibition of cancer progression, e.g., tumor necrosis factor superfamily member 14 (LIGHT), Fms-related tyrosine kinase 3 ligand (FLT-3 ligand), CXCL10, and latency-associated protein (LAP) [140,141,142]. These findings support the hypothesis that the effect of CM from MSCs could depend on its origin. Interestingly, our results also indicate that hUCESC-CM exerts no action on low-proliferating breast cancer epithelial cells (MCF7 cell line), while exerting high anti-tumor activity against highly proliferating and metastasis-producing breast cancer cells (MDA-MB-231 cell line) [23]. As previously hypothesized [143], it is not surprising that the human uterine cervix might be a reservoir of MSCs with precise site-specific functions. In fact, it is known that fundamental biological events, some of them crucial for the host’s wellbeing, take place in the cervical transformation zone. Furthermore, inflammation and regeneration are closely linked in this setting, which constitute a standard model of carcinogenesis [144]. Therefore, it can be hypothesized that hUCESCs (and their CM) have intrinsic defense mechanisms against inherent vulnerability from malignant transformation. hUCESCs might also proceed as “guardians of their environment” avoiding the acquisition of a malignant phenotype by adult cells, modulating their proliferation rate and inducing apoptosis if they become dangerous for the host.

It has also been reported that hUCESC-CM inhibits proliferation and invasion of cancer associated fibroblasts (CAFs) [21]. This is relevant because CAFs, a prevailing component of tumor stroma, contribute to tumor growth, invasion and dissemination [145,146]. CAFs promote tumor initiation and progression through the stimulation of angiogenesis, invasion, and metastasis, but also mediating drug resistance, which leads to considering them a therapeutic target in current clinical trials [147]. Another important cellular component of tumor microenvironment are tumor associated macrophages (TAMs) [148], which are a relevant inhibitor of anti-tumor immunity and a crucial barrier to successful immunotherapy [149]. In addition, TAM infiltration is associated with poor prognosis in solid tumors, including breast cancer [150,151], thus the relevance of our data showing that hUCESC-CM significantly inhibits and reverts monocyte to macrophage differentiation [21].

### 4.7. Antimicrobial Effect

Several in vivo studies have shown beneficial effects of MSC treatment in bacteria-induced sepsis [152,153,154,155,156], suggesting immunomodulatory properties of MSCs mediated by enhanced phagocyte activity. Our in vitro studies have demonstrated that hUCESC-CM have a bactericidal effect on both *E. coli* and *S. epidermidis*. In addition, hUCESC-CM has at least the same bactericidal effect as commercial solutions against *S. epidermidis* in infected contact lenses [49]. Given that these experiments were carried out in vitro and the immune response of the host cells were not involved in the process, one or more substances present in hUCESC-CM must be responsible for this antibacterial effect, such as the chemokines CXCL10, CXCL8, CXCL1, CXCL6, CCL20, and CCL5, which are present in higher levels in hUCESC-CM. These chemokines are known to have antibacterial effects against *E. coli* and different strains of *Staphylococcus* [157,158,159,160,161], which lead to consideration that a paracrine signaling might be implicated in the antibacterial potential of hUCESC-CM.

## 5. Differences in MSC-CM Composition and Need for Standardization

Although different MSC populations are known to share phenotypic characteristics and show regenerative potential, they reside in different anatomic locations and their secretome is likely to vary. Differences in therapeutic potential according to MSC origin have been demonstrated [23,162,163]. For example, while ADSC-CM is positive for HGF, VEGF, stem cell factor (SCF) and nerve growth factor (NGF), CM of human umbilical cord perivascular cells (hUCPVC-CM) only presents NGF and VEGF. Likewise, differences have been shown in the composition of ADSC-CM and adult bone marrow MSCs (BMMSC) -CM [164], as well as in secreted factors from Wharton’s jelly and BMMSCs [110]. Recently, Pires et al. [165] found important changes in the secretome of MSCs from BM, adipose tissue, and umbilical cord after a comparative proteomic based analysis by mass spectrometry. In order to standardize the production of CM from each MSC type, further studies are necessary with regard to culture medium and supplements, culture duration, and culture conditions [166]. 

## 6. Exosomes from MSCs

The term exosome generally refers to a specific class of lipid-membrane bound extracellular vesicle characterized by a diameter of 40–150 nm and a density of 1.09–1.18 g/mL. Increasing evidence indicates that MSCs produce massive amounts of exosomes in comparison with other cells, and that many of the regenerative properties previously credited to stem cells are being shown to be mediated through secreted exosomes [167]. Exosomes may subsequently be internalized by other cells principally by phagocytosis, fusion with the cell membrane and receptor–ligand interaction, allowing the release of their contents into the cytoplasm [168,169]. It has been reported that treatment with MSC-derived exosomes and microvesicles improves at least one clinically relevant parameter associated with organ functionality (Table 2). The regenerative potential of exosomes may be modulated by a variety of mechanisms, such as the prior exposure of the originating cell population to external stimuli [170,171,172]—for example, inflammatory conditioning of human umbilical cord blood-derived MSCs (hUCBSCs) with IFN-γ results in MSCs being less able to protect against acute ischemic renal injury in vivo [173] than their unconditioned counterparts.

Several specific advantages of using exosomes instead of CM for regenerative therapy have been reported [167]. Through encapsulation, therapeutically relevant molecules (proteins and nucleic acids) are protected from degradation. In this sense, MSC can be engineered to produce exosomes having the mRNA information of relevant gene in the cargo and therefore retaining the ability to home to tumor or disease site, and thus be internalized by the targeted cells [195,196]. The loading of an intracellular miRNA into exosomes could be done by engineering an extra-seed sequence (hEXO motif), which opens the way for the selective modification of the miRNA exosomal cargo [197]. In fact, it has recently been reported that in a mouse model of Huntington’s Disease, exosomes loaded with modified small interfering RNAs (hsiRNAs) targeting Huntingtin mRNA were internalized by primary cortical neurons and were able to induce mRNA and protein silencing [198]. From a manufacturing perspective, exosomes last a long time and may be stored in much smaller volumes than CM. However, potential risks associated with exosome based therapies have also been reported, such as the uncontrolled transfer of genetic information between cell populations [199]. In addition, exosomes can modulate immune response through transport and presentation of key antigens [200]. Another potential toxicity associated with exosome-based therapies is biodistribution. Bioluminescence analysis in mice of intravenously delivered, luciferase-labeled exosomes showed strong localization to the spleen, liver, lung and kidney, and possibly brain, heart and muscle within 30 min of injection, prior to spiking in the urine at 60 min post-delivery [201]. A clear understanding of the relationships between delivery site, dosage, biodistribution and clearing dynamics is essential for ensuring product biosafety [167].

Other important aspects of MSC exosomes are their tissue-specific responses by directing informational molecules to target cells, and the possibility to act as reservoir of biomarkers, which can differ depending on their sources of isolation [202]. For instance, exosomes from ADSC appear to be more effective in degrading Aβ compared to BMMSC-derived exosomes in an in vitro model of Alzheimer’s disease [203]. Likewise, it has been described that exosomes from ADSC has a therapeutic potential for treating Huntington’s Disease decreasing aggregation of mutant Huntingtin, ameliorating abnormal apoptotic protein level and reducing mitochondrial dysfunction [204]. There is greater neurite outgrowth response from exosomes released by menstrual fluid derived MSCs than from umbilical cord, chorion and BM-derived MSCs [205,206]. These findings suggest the need for further research into the functional properties of exosomes released by MSCs isolated from adult tissues of different sources, and the influence of gender on secretion profile.

## 7. Clinical Studies with Secretome from MSCs

A limited number of human clinical studies are already available on the use of secretome products from MSCs. Application of allograft ADSC-CM, after the treatment of fractional carbon dioxide laser resurfacing on human skin, enhances wound healing by the reduction of adverse effects such as hyperpigmentation, erythema and increased transepidermal water loss [61]. Likewise, intradermal injection of ADSC-CM into the scalp of alopecia patients using a split-scalp study design significantly promoted hair growth in both male and female patients [207]. Similarly, a retrospective and observational study of female pattern hair loss treated with ADSC-CM showed efficacy after 12 weeks, significantly increasing both hair density and thickness. None of the patients reported severe adverse reactions [208]. Finally, BMMSC-CM has also been used safely to improve alveolar bone regeneration [209].

Several clinical applications of MSC-derived exosomes have been reported. A preliminary study demonstrated that increasing dosage of MSC-derived exosomes in a patient with severe treatment-refractory graft-versus-host grade IV disease, affecting skin and intestinal tract, was well tolerated and showed a significant and sustainable improvement of symptoms, which remained stable for five months [210]. Another clinical trial applied MSC-derived exosomes for improving β-cell mass in type 1 diabetes mellitus patients (https://clinicaltrials.gov/ct2/show/NCT02138331?term=MSC+exosomes&rank=1). Many more studies are expected to be initiated shortly [167,210].

## 8. Scalable Production of MSC Secretome

There is presently a lack of current good manufacturing practice (cGMP) guidelines for large-scale manufacturing of MSC-derived products. It has been estimated that hundreds of micrograms to milligram of exosomes may be needed to treat patients in a clinical trial [167]. Senescence of MSCs represents an intrinsic limitation on production capacity. Cell immortalization is one approach to this issue. Indeed, an efficient strategy could be *MYC* proto-oncogene transformation to ensure an infinite supply of cells for production [211]. A bioreactor approach could eliminate the need for continuous passage of cells during production runs, thus alleviating the need for plastic vessels and medium. A combination of methodologies for isolating exosomes might also prove helpful [52].

## 9. Inducing Secretory Modifications in MSCs

There is evidence suggesting that modification of MSCs could improve the therapeutic effect of their secretome. A variety of stimuli and conditions have been advanced including: (a) cell culture under hypoxic conditions, which increases the production of growth factors and anti-inflammatory molecules; (b) pro-inflammatory stimuli, which induces higher secretion of immune-related factors; (c) tri-dimensional growth, which upregulates production of anti-tumoral and anti-inflammatory factors; and (d) microparticle engineering.

### 9.1. Hypoxia

In a variety of tissues, reduction of oxygen tension activates the hypoxia inducible factor (HIF-1α), inducing in turn, the expression of angiogenic factors such as VEGF [212,213,214]. It has been recently demonstrated that cell culture under hypoxic conditions has beneficial effects on MSCs [44]. In fact, it is known that most growth factors are upregulated in various stem cells under hypoxia conditions [166]. In addition, hypoxia allows for maintaining an undifferentiated phenotype of MSCs for self-renewal. This may be because MSCs usually are found in hypoxic areas of the body, poorly perfused by the circulatory system [213,215]. Accordingly, several studies have shown the negative influence of ambient O_2_ concentration on MSCs, inducing early senescence [216], longer population doubling time and DNA damage [217]. For example, a 3% O_2_ tension in cell culture has shown positive effects on in vitro survival and self-renewal of BMMSCs, while maintaining their undifferentiated state [218,219]. Likewise, a 2% O_2_ tension was found to preserve stemness and enhance proliferation and angiogenic potential of ADSCs [220,221]. It has recently been reported that hypoxic human dental pulp stem cells (DPSCs) are smaller in size and exhibit larger nuclei than those grown in normoxic environment. On the other hand, culture of DPSCs in 5% O_2_ significantly increases their migration and proliferation rate, but also the expression of stem cell markers and BDNF, NGF, SOX2 and VEGF [222]. All these data suggest that adequate O_2_ concentrations may enhance MSCs properties and growth, and trophic effect of their secretome. 

Treatment with hypoxic-preconditioned ADSC-CM increased the viability of hepatotoxic hepatocytes and enhanced liver regeneration in partially hepatectomized mice [223]. Therapeutic benefits of hypoxia have also been reported in a rat model of traumatic brain injury and of diabetic cardiopathy [57,224].

### 9.2. Pro-Inflammatory Stimuli

Evidence suggests that stimulation of MSCs by inflammatory factors enhances their regenerative potential and improves their anti-inflammatory response. Thus, exposure of MSCs to IFN-γ stimulates indoleamine-pyrrole 2,3-dioxygenase (IDO) enzyme production, which increases immune suppressive activity of MSCs [225,226,227]. It has also been reported that pretreatment of MSCs with TNF-α increases their angiogenic activity in vitro and in vivo in an animal model of limb ischemia [228]. Pre-treatment of MSCs with TNF-α also increases proliferation, migration, and osteogenic differentiation through up-regulation of bone morphogenetic protein-2 (BMP-2) [229]. Likewise, the regenerative activity of MSCs can be stimulated by lipopolysaccharide (LPS) or toll-like receptors (TLR) agonists through induction of paracrine factors production [230].

### 9.3. Tri-Dimensional Growth

MSCs are typically grown in vitro in monolayer systems. Nevertheless, tri-dimensional cultures, such as spheroid culture, have shown to stimulate trophic factors secretion. Spheroid cultures need special handling and equipment (spinner flask) but yield more cells compared to conventional monolayer cultures, and therefore more secreted factors [60,231]. It is worth mentioning that cells located at the center of the spheroid may be in relative hypoxic condition compared to cells on the surface. As mentioned above, oxygen concentration is a critical environmental factor that affects the maintenance of stem cell plasticity and proliferation [232]. CM from human MSC spheroids has been found to inhibit TNF-α, CXCL2, IL6, IL12p40, and IL23 production from LPS-stimulated macrophages and stimulate higher production of prostaglandin E2 (PGE2). In a murine model of zymosan-induced peritonitis, spheroids and spheroid-derived cells have more effective anti-inflammatory effects than monolayer MSC culture cells [233]. Hanging-drop-induced MSC spheres (containing 25,000 cells per drop) produced higher levels of TNF-α-stimulated gene 6 protein (TSG-6), an important anti-inflammatory factor. They also produced higher level of stanniocalcin-1 (STC1), an anti-inflammatory and antiapoptotic protein, as well as three anticancer proteins [233]. Dynamic cultures using spinner flasks or rotating wall vessel bioreactors form small spheroids and have demonstrated better adipogenic and osteogenic differentiation and also a higher expression of IL24 [234].

### 9.4. Microparticle Engineering

Certain properties of CM can be modified via microparticle engineering. It has recently been reported that microparticles loaded with 2-[(aminocarbonyl)amino]-5-(4-fluorophenyl)-3-thiophenecarboxamide (TPCA-1), an NF-κB inhibitor, can attenuate the secretion by MSCs of pro-inflammatory factors for at least six days in vitro. CM derived from TPCA-1-loaded MSCs also showed reduced ability to attract human monocytes and prevented differentiation of human cardiac fibroblasts to myofibroblasts [235]. This is an interesting finding because adverse remodeling or cardiac fibrosis due to differentiation of cardiac fibroblasts into myofibroblasts with pro-inflammatory phenotype and collagen deposition is the leading cause for heart failure. 

## 10. Conclusions

MSCs have drawn much interest for their therapeutic benefits in immune modulation and tissue remodeling. Earlier research primarily attributed this to the ability of transplanted MSCs to engraft and to differentiate into multiple tissue types. However, there are several limitations that currently impair the wide widespread use of MSCs. Some studies suggest that the transportation of MSCs into normal tissues might cause tumor formation. Recent studies have revealed that implanted MSCs do not survive for long, suggesting that the benefits of MSC therapy might be attributable to their secreted factors. An expanding body of recent literature has brought attention to the vast array of bioactive factors produced by MSCs including cytokines, growth factors, microRNAs, proteasomes, and exosomes, which may play an important role in the regulation of numerous physiological processes. Secretome-based approaches using CM or exosomes may present considerable potential advantages over living cells in terms of manufacturing, storage, handling, product shelf life and their potential as a ready-to-go biological therapeutic agent. Multiple experimental studies demonstrate that secretome-derived products are sufficient to significantly improve multiple biomarkers of pathophysiology in many animal models of different diseases. However, composition and effects of secretomes differ based on MSC anatomical origin. Using hUCESCs, in particular, presents important advantages. hUCESCs are obtained by Pap cervical smear, which is a less invasive and painful method than those used for obtaining other MSCs (e.g., bone marrow or adipose tissue). In addition, due to their easy isolation and high proliferative rate, it is possible to quickly obtain large amounts of hUCESCs or secretome-derived products for research and clinical use.

Considering that multiple clinical trials involving MSCs have been approved by national agencies, it is perfectly reasonable to expect approval of secretome-derived products from MSCs. Although the composition of secretome-derived products from MSCs is complex, this in itself should not be an impediment for regulatory approval of a regenerative product. For example, platelet-rich plasma or amniotic fluid, which are highly complex and include numerous growth factors and exosomes that remain poorly characterized, are routinely used as a regenerative therapy for multiple applications in wound healing and orthopedics. Exosomes sourced from dendritic cells have already reached the clinical-trial stage for immunotherapy of certain cancers. Nevertheless, regulatory requirements for manufacturing and quality control will be necessary to establish the safety and efficacy profile of these products.

## Figures and Tables

**Table 1:** Studies involving the use of mesenchymal stem cells-conditioned medium (MSC-CM).

Pathologies	Donor cells	References
Lung injury	BMMSCs	[55]
Myocardial infarction	ADSCs	[56]
Cerebral injury/ischemia/stroke	BMMSCs	[57]
Spinal cord injury	BMMSCs	[58]
Prevention of muscular degeneration	ADSCs	[59]
Acute and chronic hind limb ischemia	ADSCs	[60]
Skin wound healing	ADSCs	[61]
Colitis	Amniotic fluid	[62]
Acute liver injury/failure	Amniotic fluid	[63]
Alzheimer’s disease	DPSCs	[64]
Bone defects	BMMSCs	[65]
Osteoarthritis	WJMSCs	[66]
Corneal epithelial wound healing	hUCESCs	[49]
Uveitis	hUCESCs	[50]
Alopecia	ADSCs	[67]
Liver fibrosis	UCPVCs	[68]
Parkinson’s disease	WJMSCs	[69]
Multiple esclerosis	PDLSCs	[70]
Regeneration of atrophied muscles	UCPVCs	[71]
Cancer	hUCESCs	[23]

BMMSCs: Bone marrow-derived mesenchymal stem cells; ADSCs: Adipose-derived stem cells; DPSC: Dental pulp stem cells; WJMSCs: Umbilical cord Wharton's Jelly mesenchymal stem cells; hUCESCs: Human uterine cervical stem cells; UCPVCs: Umbilical cord perivascular cells; PDLSCs: Periodontal ligament stem cells.

**Table 2:** Beneficial effects of MSC-derived exosomes and microvesicles.

Parameters Improved	Donor Cells	Exosomes/Microvesicles	References
Decreased infarct size in myocardial ischemia/reperfusion injury	BMMSCs	Exosomes	[174]
Renoprotective effects in acute kidney disease	BMMSCs	Microvesicles	[175]
Improvement of pulmonary inflammation in acute lung injury	BMMSCs	Microvesicles	[176]
Cutaneous regeneration in wound healing	UCPVCs	Exosomes	[177]
Attenuated acute pancreatitis	BMMSCs	Microvesicles	[178]
Alleviated liver fibrosis	UCPVCs ADSCs	Exosomes	[179] [180]
Hepatic regeneration in liver injury	BMMSCs	Exosomes	[181]
Blockage of experimental autoimmune encephalomyelitis	PDLSCs	Exosomes	[70]
Delayed occurrence of graft-versus-host disease	UCPVCs	Exosomes	[182]
Suppression of tumor progression and angiogenesis	BMMSCs	Exosomes	[183]
Promotion of breast cancer cell dormancy in a metastatic niche	BMMSCs	Exosomes	[184]
Rescue of bone marrow MSC function in lupus	BMMSCs	Exosomes	[185]
Amelioration of experimental autoimmune uveitis	UCPVCs	Exosomes	[186]
Protected ischemic myocardium from ischemia/reperfusion injury	ADSCs	Exosomes	[187]
Prevent abnormal neurogenesis and memory dysfunction in epilepsy	BMMSCs	Exosomes	[188]
Stimulated chondrocyte migration and proliferation in osteoarthritis	Sinovial membrane	Exosomes	[189]
Increased tumor death in glioblastoma	BMMSCs	Exosomes	[190]
Increased chemosensitivity in hepatocellular carcinoma	ADSCs	Exosomes	[191]
Attenuated bladder tumor cell growth	WJMSCs	Microvesicles	[192]
Inhibited Kaposi’s sarcoma growth	BMMSCs	Microvesicles	[193]
Inhibited hepatoma growth	Liver	Microvesicles	[194]

## Abbreviations

ADSCs	Adipose-derived stem cells
BMMSCs	Bone marrow-derived mesenchymal stem cells
CM	Conditioned medium
DPSCs	Dental pulp stem cells
hUCESCs	Human uterine cervical stem cells
UCPVCs	Umbilical cord perivascular cells
MSCs	Mesenchymal stem cells
NSCs	Neural stem cells
PDLSCs	Periodontal ligament stem cells
SHED	Stem cells from human exfoliated deciduous teeth
UCBSCs	Umbilical cord blood stem cells
WJMSCs	Umbilical cord Wharton’s Jelly mesenchymal stem cells
